# Association of proton pump inhibitor use with survival and adverse effects outcomes in patients with multiple myeloma: pooled analysis of three clinical trials

**DOI:** 10.1038/s41598-023-48640-1

**Published:** 2024-01-05

**Authors:** Sara A. Almansour, Mohammad A. Y. Alqudah, Ziad Abuhelwa, Humaid O. Al-Shamsi, Mohammad H. Semreen, Yasser Bustanji, Nelson C. Soare, Ross A. McKinnon, Michael J. Sorich, Ashley M. Hopkins, Ahmad Y. Abuhelwa

**Affiliations:** 1 Department of Pharmacy Practice and Pharmacotherapeutics, University of Sharjah, Sharjah, United Arab Emirates; 2 Department of Clinical Pharmacy, Faculty of Pharmacy, Jordan University of Science and Technology, Irbid, 22110 Jordan; 3 Department of Hematology and Medical Oncology, University of South Florida/ H. Lee Moffitt Cancer Center and Research Institute, Tampa, FL 33612 USA; 4 Department of Oncology, Burjeel Cancer Institute, Burjeel Medical City, P.O. Box 92510, Abu Dhabi, United Arab Emirates; 5 Emirates Oncology Society, P.O.Box: 6600, Dubai, United Arab Emirates; 6 Gulf Medical University, P.O. Box: 4184, Ajman, United Arab Emirates; 7 Gulf Cancer Society, P.O. Box 26733, 13128 Alsafa, Kuwait; 8 Research Institute of Medical and Health Sciences, University of Sharjah, Sharjah, United Arab Emirates; 9 Department of Medicinal Chemistry, University of Sharjah, Sharjah, United Arab Emirates; 10 College of Medicine, University of Sharjah, P. O. Box 27272, Sharjah, United Arab Emirates; 11 School of Pharmacy, The University of Jordan, Amman, Jordan; 12 College of Medicine and Public Health, Flinders University, Bedford Park, SA Australia

**Keywords:** Cancer, Myeloma, Combination drug therapy

## Abstract

Proton pump inhibitors (PPIs) are commonly used in cancer patients, but their impact on treatment outcomes in multiple myeloma (MM) patients remains unclear. This study investigated the association of PPI use with survival and adverse effects in MM patients across three randomized-control trials initiating daratumumab, lenalidomide, or bortezomib combination treatments. Cox proportional hazard analysis and logistic regression were employed to assess the associations with treatment outcomes, while adjusting for age, sex, weight, MM international staging system stage, ECOG-performance status, comorbidity count, and presence of gastrointestinal disorders. Pooled data involving 1804 patients revealed that 557 (32%) used PPIs at baseline. PPI use was independently associated with worse overall survival (adjusted HR [95% CI] 1.32 [1.08–1.62], *P* = 0.007) and grade ≥ 3 adverse events (adjusted OR [95% CI] 1.39 [1.03–1.88], *P* = 0.030). However, the association with progression-free survival did not reach statistical significance (adjusted HR [95% CI] 1.14 [0.97–1.33], *P* = 0.112). Findings were consistent across trials and treatment arms. PPI use was identified as a negative prognostic factor in MM patients, potentially enhancing clinical decisions regarding its use. Further research is needed to fully comprehend the impacts and safety of PPI use in MM patients.

## Introduction

Multiple myeloma (MM) is the second most common haematological cancer and is characterized by an abnormal proliferation of clonally transformed plasma cells within the bone marrow^1^. Over the past decade, the treatment of MM has witnessed remarkable advancements, resulting in improved patient outcomes and prolonged survival.

Among the various advancements, the combination of immunomodulatory agents such as lenalidomide or proteasome inhibitors like bortezomib, with dexamethasone, have emerged as standard frontline therapy options for patients with MM^1,2^. Additionally, the introduction of monoclonal antibodies targeting CD38, such as daratumumab, have further enhanced available options^1^. However, despite these advancements, the treatment of MM is still associated with considerable heterogeneity in survival outcomes, adverse effects, and likelihoods of treatment resistance and failure^3^. This highlights the need for continued exploration of factors that can predict likely outcomes and aid in the selection of treatment.

Concomitant medications are commonly used in patients with cancer to manage comorbidities and treatment-related side effects. Proton pump inhibitors (PPIs) are amongst the most widely prescribed drugs, due to the frequency at which patients with cancer experience gastrointestinal (GI) diseases—such as gastroesophageal reflux disease (GERD) and peptic ulcers^4^. However, in combating these symptoms, PPIs may disrupt the gut microbiota, increase susceptibility to infections, and potentially interfere with the dissolution of orally administered medicines. Such impacts have significant potential to affect the gut-immune axis and pharmacokinetic exposures to anticancer medicines, which in turn has the potential to impact the likely survival outcomes of patient with cancer^5–11^.

Understanding the potential impact of PPIs in patients with cancer is of great clinical importance as research has demonstrated that while they are often necessary, up to 60% of myeloma patients received PPI prophylaxis during and beyond anticancer therapy without an accepted indication^12^ (i.e., PPIs are frequently overprescribed due to a presumption that they will not cause any negative impacts). Notably, much recent research indicates that PPIs are likely associated with significant changes in the efficacy of immune checkpoint inhibitors used in the treatment of solid tumours^13–15^. Yet, despite the immunomodulatory foundations of many agents used in the treatment of MM, the relationship between PPI use and survival outcomes remains largely unexplored in patients with this disease. This study aimed to investigate the association of PPI use with survival outcomes and the incidence of grade ≥ 3 adverse events in patients with MM.

## Methods

### Patient population

Individual patient data was pooled from 3 randomized, open-label trails: MAIA (NCT02252172, data cut-off: February 19, 2021)^16^, POLLUX (NCT02076009, data cut-off: March 7, 2016)^17^, and CASTOR (NCT02136134, data cut-off: January 11, 2016)^18^.

All studies enrolled adult patients aged 18 years or older. POLLUX and CASTOR assessed the efficacy of daratumumab on patients with relapsed or refractory MM who had received at least one prior line of therapy. The MAIA trial included newly diagnosed MM patients who were not eligible for high dose chemotherapy or autologous stem cell transplantation due to age (≥ 65 years) or the presence of coexisting conditions that may result in unacceptable side effects^18^.

In the MAIA and POLLUX trials, daratumumab was administered as a 16 mg/kg intravenous (IV) infusion in combination with lenalidomide (25 mg capsule orally) and dexamethasone (40 mg orally or intravenously) (DRd) compared to lenalidomide plus dexamethasone (Rd). In the CASTOR trial, daratumumab (16 mg/kg IV infusion) was administered in combination with bortezomib (1.3 mg/m^2^ subcutaneously) and dexamethasone (20 mg orally) (DVd) compared to bortezomib plus dexamethasone alone (Vd).

All studies were conducted in accordance with the International Conference on Harmonisation Good Clinical Practice guidelines and the Declaration of Helsinki. Participants provided written informed consent. The secondary analysis of de-identified data reported in this study was considered negligible risk research and has been approved by the University of Sharjah Ethics Committee (Approval reference number: REC-23-11-07-01-F). Data were accessed according to the Johnson & Johnson policy and made available through Vivli, Inc. (www.vivli.org).

### Outcome and predictor data

Within each of MAIA, POLLUX, and CASTOR progression free survival (PFS) was defined as the time from patient randomization to either disease progression according to the international myeloma working group (IMWG) response criteria or death, whichever occurred first. Overall survival (OS) was defined as the time from the date of randomization to the date of the participant’s death.

Documented use of PPI at baseline (i.e. at the screening visit/prior to treatment initiation) was the primary covariate in this study. Analyses were adjusted for age, sex, weight, MM international staging system (ISS) stage, Eastern Cooperative Oncology Group performance status (ECOG-PS) score, comorbidity count, and presence of gastrointestinal disorders (e.g., GERD, peptic ulcer disease). The rationale behind the selection of the adjustment variables is provided in Supplementary Table 1. Missing data was imputed via the Transcan function in the Hmisc (version 5.1-0) R package. Transcan is a nonlinear additive transformation and imputation function^19^.

### Statistical analysis

Cox proportional hazards regression was employed to examine the associations between PPI use and OS/PFS. The assessment of PPI independence from other prognostic factors was evaluated using univariate and adjusted analyses. Results were reported as hazard ratios (HR) with 95% confidence intervals (95% CI). Statistical significance was set at *P*-value < 0.05. All models were stratified by clinical trial and treatment arms to account for potential variations between the studies and treatment approaches. Heterogeneity of PPI associations were assessed according to study and treatment interaction analyses. Forest plots were utilized to visually present the HRs (and 95% CI) of subgroups for conducted interaction analyses. Kaplan–Meier plots were employed to graphically depict and estimate survival probabilities based on PPI use. The association between PPI use and any grade ≥ 3 adverse events, occurring within the first 12 months of treatment initiation, was assessed using logistic regression analysis. The results were reported as odds ratios (ORs) along with their corresponding 95% CI. All analyses were performed using R version 4.2.

### Ethics approval

Secondary analysis of anonymised clinical-trial data was confirmed as negligible-risk research and has been approved by University of Sharjah Research and Ethics Committee (Approval reference number: REC-23-11-07-01-F).

## Results

### Patient population

The pooled cohort consisted of 1804 patients, of whom 557 (32%) received PPI at baseline. A summary of patients’ baseline characteristics by study is provided in Table 1 and a summary of baseline chatacteristics by PPI use is provided in Supplementary Table 2. The median follow-up time was 56.2 months for the MAIA, 7.43 months for the CASTOR, and 13.5 months for the POLLUX study.

**Table 1:** A summary of Patients' baseline characteristics by study.

Variable	Total no. 1804	CASTOR no. 498	MAIA no. 737	POLLUX no. 569	*P*-value
Arm of the clinical study					< 0.001
Bortezomib and dexamethasone	247 (14%)	247 (50%)	0 (0%)	0 (0%)	
Daratumumab plus bortezomib and dexamethasone	251 (14%)	251 (50%)	0 (0%)	0 (0%)	
Daratumumab plus lenalidomide and dexamethasone	654 (36%)	0 (0%)	368 (50%)	286 (50%)	
Lenalidomide and dexamethasone	652 (36%)	0 (0%)	369 (50%)	283 (50%)	
Age (years)	66 (58–72)	58 (48–68)	72 (64–72)	65 (59–71)	< 0.001
Sex					0.029
Male	1005 (56%)	284 (57%)	384 (52%)	337 (59%)	
Female	799 (44%)	214 (43%)	353 (48%)	232 (41%)	
Weight (kg)	73 (63–85)	76 (67–88)	72 (63–84)	72 (61–84)	< 0.001
Race					< 0.001
White	1516 (84%)	436 (88%)	677 (92%)	403 (71%)	
Asian	132 (7%)	24 (5%)	5 (1%)	103 (18%)	
Black or African American	83 (5%)	23 (5%)	34 (5%)	26 (5%)	
Other	73 (4%)	15 (3%)	21 (3%)	37 (7%)	
Proton Pump Inhibitor (Y/N)	577 (32%)	143 (29%)	264 (36%)	170 (30%)	0.014
ISS disease stage					< 0.001
I	672 (37%)	194 (39%)	201 (27%)	277 (49%)	
II	692 (38%)	194 (39%)	319 (43%)	179 (31%)	
III	440 (24%)	110 (22%)	217 (29%)	113 (20%)	
ECOGPS					< 0.001
0	761 (42%)	222 (45%)	250 (34%)	289 (51%)	
1	863 (48%)	244 (49%)	365 (50%)	254 (45%)	
≥ 2	180 (10%)	32 (6%)	122 (17%)	26 (5%)	
Comorbidity count	5.0 (3.0–7.0)	4.0 (2.0–6.0)	6.0 (4.0–8.0)	4.0 (3.0–6.0)	< 0.001
Gastrointestinal disorders	765 (42%)	157 (32%)	380 (52%)	228 (40%)	< 0.001
Adverse events (grade ≥ 3)	1469 (81%)	347 (70%)	681 (92%)	441 (78%)	< 0.001

Data are median (IQR) or number of patients (%).

*ISS Stage* International staging system (ISS) stage, *ECOGPS* Eastern Cooperative Oncology Group performance status.

*P* values per Chi-Square test for categorical data and Kruskal–Wallis test for continuous data.

Regarding missing data, patient weight was missing for 39 (8%) patients in CASTOR and 287 (50%) in POLLUX. For the ECOG score, only 1 patient (< 1%) had missing data in CASTOR, and race had missing data in 11 (2%), 16 (2%), and 57 (10%) patients in CASTOR, MAIA, and POLLUX, respectively. All other variables had complete data. Supplementary Table 3 provides a summary of un-imputed patients' baseline characteristics by study.

Among the patients using PPIs, 335 (58%, *P* < 0.001) had a GI disorder documented in their medical history. While the lower levels details on the GI disorders were not provided in the MAIA trial data, within CASTOR and POLLUX it was observed that the most frequent GI disorders associated with PPI were gastroesophageal reflux disease (GERD) (n = 45, 13.4%), ulcers encompassing duodenal and/or gastric ulcer (n = 11, 3.2%), gastritis (n = 18, 5.3%), hernia (n = 33, 9.8%), and others (n = 55, 16.4%) including esophagitis, acid peptic disease, dyspepsia, dysphagia, and gastric polyps.

In CASTOR and POLLUX, PPI use was documented by class name—‘proton pump inhibitors’. Within MAIA, it was noted that PPI use (n = 264) related to the specific use were pantoprazole (n = 86, 33%), omeprazole (n = 75, 28%), esomeprazole (n = 65, 25%), lansoprazole (n = 26, 10%), dexlansoprazole (n = 6, 2%), and rabeprazole (n = 6, 2%).

Grade ≥ 3 adverse events occurred in 1469 (81%) patients, of whom 502 (87%) were PPI users (Supplementary Table 2). The top ten most common grade ≥ 3 adverse events by study are represented in the Supplementary Table 4. Notably, neutropenia was the most common adverse event across all cohorts, affecting 634 (35%), followed by thrombocytopenia in 336 (19%) and anaemia in 331 (18%) patients.

### PPI use and survival outcomes

**Figure 1:**
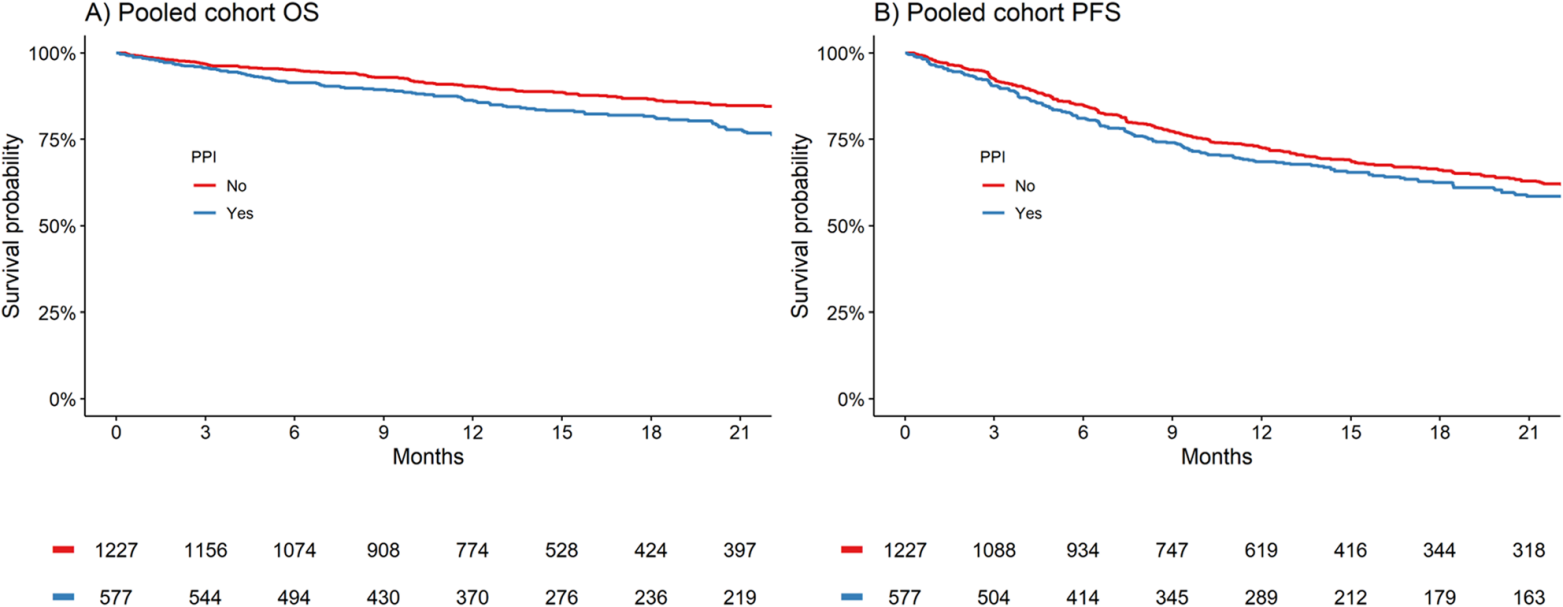
Kaplan–Meier curves for PPI use and outcomes in pooled cohort. Kaplan Meier estimates for pooled cohort (**A)** overall survival and (**B)** progression free survival.

The prognostic associations between PPI use and survival outcomes are presented in Table 2. In pooled univariable analyses, PPI use was statistically associated with worse OS (HR [95% CI] 1.49 [1.22–1.81], *P* < 0.001) and PFS (HR [95% CI]  1.19 [1.02–1.39], *P* = 0.03) outcomes. Similarly, in adjusted analyses, PPI use remained statistically associated with worsened OS (HR 1.32, 95% CI 1.08–1.62, *P* = 0.007). However, the association between PPI use and PFS did not reach statistical significance (adjusted HR 1.14, 95% CI 0.97–1.33, *P* = 0.1). Kaplan–Meier estimates for the survival outcomes by PPI use are depicted in Fig. 1.

**Table 2:** Univariate and adjusted pooled analysis of the association of PPI use with survival and adverse events grade ≥ 3 outcomes.

Pooled	Events/Subj	Univariate		Adjusted^a, b^	
PPI (Y)		HR (95% CI)	*P*-value	HR (95% CI)	*P*-value
OS	174/577	1.49 [1.22–1.81]	< 0.001	1.32 [1.08–1.62]	0.007
PFS	255/577	1.19 [1.02–1.39]	0.03	1.14 [0.97–1.33]	0.1
Adverse events (grade ≥ 3)^c^	502/577	1.69 [1.27–2.26]	< 0.001	1.39 [1.03–1.88]	0.030

^a^Analyses stratified by study and arms.

^b^Analysis adjusted for PPI, age, sex, ISS disease stage, weight, ECOG score, comorbidity count, and presence of gastrointestinal disorders.

^c^Results are odds ratios (OR) obtained from logistic regression analysis.

**Figure 2:**
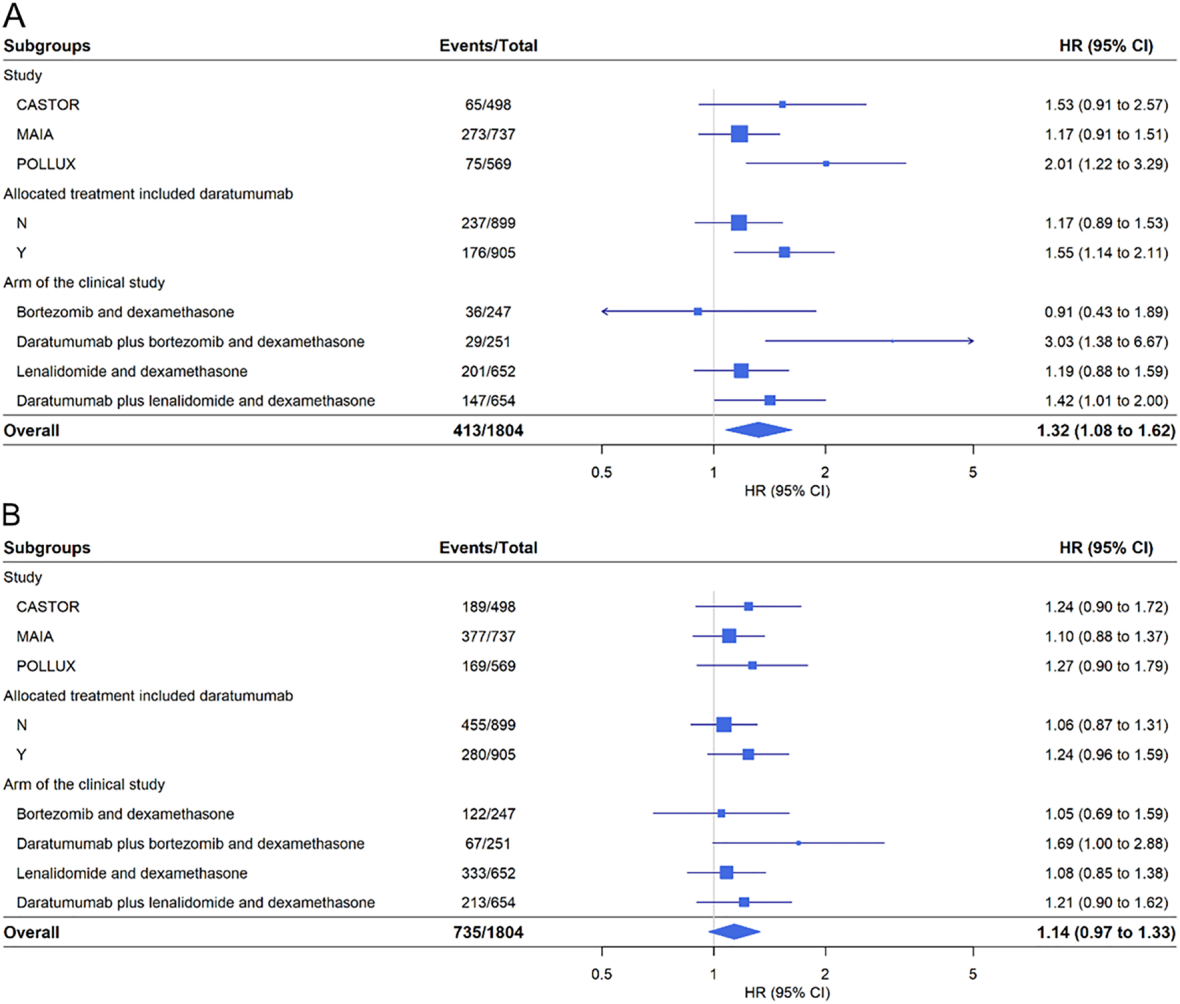
Adjusted subgroup analysis for OS (**A**) and PFS (**B**) by study, daratumumab arms, and treatments arms. Adjustment variables included age, sex, ISS disease stage, weight, ECOG score, comorbidity count, and presence of gastrointestinal disorders.

There was no statistically significant differences in the observed associations between PPI use with either OS (*P*-interaction = 0.2) or PFS (*P*-interaction = 0.8) between clinical trials, nor between treatment arms (PFS *P*-interaction = 0.7; OS *P*-interaction = 0.4). The estimated adjusted HRs (and 95% CI) for the interaction analyses by clinical trial and treatment arms are presented in Fig. 2. PPI use was associated with worse OS outcomes for the pooled estimate (HR [95% CI]  1.32 [1.08–1.62]). Notably, treatment arms incorporating daratumumab exhibited a significant association with worse survival outcomes in both DVd (HR [95% CI]  3.03 [1.38–6.67]) and DRd (HR [95% CI]  1.42 [1.01–2.00]). There was no significant association for treatment arms without daratumumab, however they were trending towards worse outcomes. PPI use did not show a significant association with the pooled estimate for PFS (HR [95% CI] 1.14 [0.97–1.33]). Subgroup specific Kaplan–Meier plots are presented in Supplementary Fig. 2.

### *PPI use and grade* ≥ *3 adverse events*

The association between PPI use and grade ≥ 3 adverse events is presented in Table 2. PPI use was statistically significant with patients experiencing adverse events of grade ≥ 3 for both the univariate and multivariate analyses (OR [95% CI] 1.69 [1.27–2.26], *P* < 0.001) and (adjusted OR [95% CI] 1.39 [1.03–1.88], *P* = 0.030).

## Discussion

This study provides insights about the association of baseline PPI use with survival and adverse events grade ≥ 3 outcomes in MM patients across three distinct cohorts. The findings indicate a significant association between PPI use and worse OS and grade ≥ 3 adverse events across all cohorts. PFS did not show a significant association; however, it was trending towards worse outcomes. To the best of our knowledge, this study is the first to comprehensively examine the relationship between PPI use and outcomes in MM patients receiving multi-drug immunomodulatory combinations. Notably, PPI use was identified as a negative prognostic factor regardless of the study cohort or treatment arm, suggesting a consistent association between PPI use and worse outcomes.

Accumulating evidence links PPI use and increased mortality rates in cancer. A recent study on hematologic malignancies, including MM, revealed significantly higher hazard for cancer-specific mortality (adjusted HR 1.31, 95% CI 1.18–1.44) and 1-year cancer-specific mortality (adjusted HR 1.50, 95% CI 1.29–1.74) in PP users^20^. Despite differences in outcome measures, these findings align with our results, indicating a negative association between PPI use and survival outcomes. Additionally, studies on solid tumours such as non-small cell lung cancer (NSCLC) and colorectal cancer have similarly demonstrated unfavourable prognostic effects associated with PPI use on survival^9,15,21–23^. Nevertheless, some studies suggest positive correlation between PPI use and cancer outcomes. In a study on untreated head and neck squamous carcinoma patients, the use of PPIs or histamine-2 receptor antagonists (H2Ras), either alone or in combination, was associated with significantly improved overall survival^24^. However, a limitation of this study was the absence of randomization. Additionally, an experimental study on human MM cells reported that lansoprazole exerted a direct antitumor effect through direct cytotoxicity and apoptotic-like cell death^25^. However, it is crucial to consider that the study was conducted in vitro and may not fully represent PPI effects in vivo.

Recently, there has been growing evidence of the role of the gut microbiome on various diseases, including MM. PPIs can alter the gut microbiome, causing gut dysbiosis by reducing gastric acid secretion^8^. Several studies reported reduced gut microbiota diversity and an increase in Streptococcoceae, Micrococcoceae, and Enterococcoceae in PPI users versus non-users^7,26^. Additionally, PPIs have been linked to higher risks of Clostridium difficile infections and colonization by drug-resistant organisms, potentially contributing to adverse health outcomes^8^. Although the precise ways the gut microbiome affects the host systems are not fully elucidated, it impacts processes crucial to hematological malignancies, such as micronutrient processing and immune system activation^8,27,28^. In a study comparing MM patients to healthy controls, alterations in the gut microbiome were found to actively contribute to MM progression. MM patients exhibited higher levels of nitrogen-recycling bacteria like Klebsiella and Streptococcus that hydrolyse urea for the synthesis of L-glutamine, a key factor in myeloma progression^27,28^. Furthermore, in MM mouse models, the presence of Prevotella heparinolytica in the gut influenced the immune system through T-helper 17 cells, which causes T cells to migrate to the myeloma environment and fuel tumor progression through IL-17 production^29^. Growing concern surrounds the impact of PPIs on the efficacy of anti-cancer drugs including immunotherapy and monoclonal antibodies (mAb), whether administered orally or intravenously. A retrospective study highlighted increased adverse events when PPIs were used concomitantly with the mAb’s cetuximab and panitumumab^4^. As for immunotherapy, multiple studies reported of PPIs affecting drug efficacy and survival outcomes of patients receiving ICIs^15^ and anti-PD-1/PD-L1 therapies^30^. Although existing literature doesn't confirm drug interactions between PPIs and daratumumab or lenalidomide, our findings suggest consistent unfavorable outcomes across different treatments, implying a persistent negative association with PPI use irrespective of the therapy employed.

Both lenalidomide and daratumumab operate by mechanisms that rely on the immune system. Lenalidomide can alter cytokine production, regulate T cell co-stimulation, and enhance natural killer (NK) cell-mediated cytotoxicity^31,32^, while daratumumab induces antibody dependent cell-mediated cytotoxicity (ADCC) and the antibody-dependent cellular phagocytosis (ADCP)^33^. Hence, interactions between PPIs, the gut microbiota, or the immune system may potentially influence the efficacy of these drugs. In addition to gut dysbiosis, PPIs may also promote T cell tolerance^34^ and affect immune cell functions. A study on omeprazole revealed that it significantly reduces NK cell functions and cytotoxicity at normal therapeutic doses (20 mg/d)^35^.

Emerging evidence has highlighted the association between PPI use and various serious adverse events. These include gastrointestinal and extraintestinal complications such as pneumonia, electrolyte imbalances, and vitamin deficiency^25,36^. Additionally, long-term PPI use is associated with reduced red and white blood cell counts, hemoglobin levels, iron deficiency, and risk of osteoporosis^37,38^. Some studies also reported of PPI-induced thrombocytopenia or neutropenia^39–41^. These potential adverse events are of great clinical implications for cancer patients, underscoring the need for further research to comprehensively understand the safety profile of PPIs and their potential impact on patients with cancer.

Limitations of this study include the lack of information regarding the specific type and duration of PPI use, hampering the assessment of their potential association with treatment outcomes. Additionally, generalizing study findings to the real-world population is constrained by eligibility criteria applied in clinical trials. For instance, exclusion of patients with smoldering MM or primary amyloidosis and those with prior anti-CD38 therapies or stem cell transplantation, limits broader applicability. Additionally, the evaluation of daratumumab-based combinations in specific patient populations, like refractory or relapsed MM in POLLUX and CASTOR, and newly diagnosed MM in MAIA, may not fully represent the diversity of patients in real-world settings. Another potential limitation is the completeness of the data. Despite relatively low missing data percentages and the use of imputation methods to minimize uncertainty, some bias possibility remains.

In conclusion, this study identified a significant association between PPI and worse OS outcomes and increased odds of experiencing grade ≥ 3 adverse events within a pooled cohort of MM patients treated with contemporary treatment options. These findings may potentially optimize patient care and improve clinicians’ decision-making in prescribing PPIs. This may involve avoiding unnecessary use or considering the shortening of their usage as clinically indicated, given that presuming that they are completely harmless may potentially be inappropriate. However, we also acknowledge the inherent limitations associated with using clinical trial data and these findings need to be validated using real world data. It is also important to investigate whether these associations extend to treatment options beyond daratumumab, lenalidomide, bortezomib, and dexamethasone, and whether the associations become apparent with respect to PFS in a larger cohort. Further research is warranted to elucidate the underlying mechanisms of these associations.

### Supplementary Information

Supplementary Information.

## Data Availability

Data were accessed according to YODA policy and process for clinical study data sharing and is available for request at https://yoda.yale.edu/.
