# Supplementary material for: Association of proton pump inhibitor use with survival and adverse effects outcomes in patients with multiple myeloma: pooled analysis of three clinical trials
Source: Sci Rep. 2024 Jan 5;14:591. doi: 10.1038/s41598-023-48640-1 (PMC10770405; doi:10.1038/s41598-023-48640-1)
Supplement: Supplementary file 1 — Supplementary Information. [file 41598_2023_48640_MOESM1_ESM.docx]

# Supplementary material

Supplementary Table 1. Rationale for selecting adjustment variables

| **Variable** | **Rationale** | **Reference** |
| --- | --- | --- |
| **Age** | Prior studies suggest that age is a significant independent prognostic factor for overall survival in MM patients. Patients who are >50 years of age display significantly shorter median survival times than younger patients. | [1-2] |
| **Sex** | Gender may possibly influence primary genetic events of MM that could affect survival outcomes. Prior evidence suggests inferior overall survival in females compared to males. This may be due to the higher prevalence of lesions associated with poor prognosis in females such as t(4;14), t(14;16) and +1q. However, other studies report of worse survival outcomes for males. | [3-5] |
| **Weight** | Several studies have highlighted the effect of BMI on MM patients and its association with survival outcomes. For instance, a study suggests that being underweight or having severe obesity is associated with worse survival. While another study reported that female patients with elevated BMI had enhanced survival, whereas a higher BMI was associated with inferior OS among male patients. | [6-8] |
| **ISS stage** | ISS staging has been reported as an indicator for overall survival in MM patients. Stage II or III are associated with worse outcomes. | [2, 4, 9] |
| **ECOG-PS score** | ECOG performance score that is >= 2 has been associated with inferior OS and PFS in MM patients. | [4, 10] |
| **Comorbidity count** | The presence of comorbidities in MM patients is significantly associated with an increased risk of mortality. A study reported of increased risk of death for patients with one comorbidity at diagnosis compared to those without any comorbidity. The risk was higher for those with two or more comorbidities. | [11-12] |
| **Gastrointestinal disorders** | Patients with concurrent gastrointestinal disorders for which PPIs are indicated such as ulcers may have different comorbidity profiles that lead to a higher baseline risk of mortality. A study on MM patients reported of the association between worse survival outcomes and various comorbidities including ulcer disease. | [13] |

MM: Multiple myeloma, OS: Overall survival, PFS: Progression free survival, ISS Stage: international staging system (ISS) stage, ECOGPS: Eastern Cooperative Oncology Group performance status.

Supplementary Table 2. A summary of patients’ baseline characteristics by Proton Pump Inhibitor use

| **Variable** | **Total No. 1,804** | **No No. 1,227** | **Yes No. 577** | **P-value** |
| --- | --- | --- | --- | --- |
| **Study** | | | | 0.014 |
| CASTOR | 498 (28%) | 355 (29%) | 143 (25%) |  |
| MAIA | 737 (41%) | 473 (39%) | 264 (46%) |  |
| POLLUX | 569 (32%) | 399 (33%) | 170 (29%) |  |
| **Arm of the clinical study** | | | | 0.33 |
| Bortezomib and dexamethasone | 247 (14%) | 176 (14%) | 71 (12%) |  |
| Daratumumab plus bortezomib and dexamethasone | 251 (14%) | 179 (15%) | 72 (12%) |  |
| Daratumumab plus lenalidomide and dexamethasone | 654 (36%) | 435 (35%) | 219 (38%) |  |
| Lenalidomide and dexamethasone | 652 (36%) | 437 (36%) | 215 (37%) |  |
| **Age (years)** | 66 (58 - 72) | 64 (58 - 72) | 68 (64 - 72) | < 0.001 |
| **Sex** | | | | 0.69 |
| Male | 1,005 (56%) | 688 (56%) | 317 (55%) |  |
| Female | 799 (44%) | 539 (44%) | 260 (45%) |  |
| **Weight (kg)** | 73 (63 - 85) | 73 (63 - 85) | 74 (64 - 85) | 0.23 |
| **Race** | | | | 0.021 |
| White | 1,516 (84%) | 1,012 (82%) | 504 (87%) |  |
| Asian | 132 (7%) | 103 (8%) | 29 (5%) |  |
| Black or African American | 83 (5%) | 56 (5%) | 27 (5%) |  |
| Other | 73 (4%) | 56 (5%) | 17 (3%) |  |
| **ECOGPS** | | | | < 0.001 |
| 0 | 761 (42%) | 561 (46%) | 200 (35%) |  |
| 1 | 863 (48%) | 567 (46%) | 296 (51%) |  |
| ≥2 | 180 (10%) | 99 (8%) | 81 (14%) |  |
| **ISS disease stage** | | | | 0.006 |
| I | 672 (37%) | 486 (40%) | 186 (32%) |  |
| II | 692 (38%) | 461 (38%) | 231 (40%) |  |
| III | 440 (24%) | 280 (23%) | 160 (28%) |  |
| **Comorbidity count** | 5.0 (3.0 - 7.0) | 4.0 (3.0 - 6.0) | 6.0 (4.0 - 7.0) | < 0.001 |
| **Gastrointestinal disorders** | 765 (42%) | 430 (35%) | 335 (58%) | < 0.001 |
| **Adverse events (grade ≥3)** | 1,469 (81%) | 967 (79%) | 502 (87%) | < 0.001 |

Data are median (IQR) or number of patients (%). P values per Chi-Square test for categorical data and Kruskal-Wallis test for continuous data. ISS Stage: international staging system (ISS) stage, ECOGPS: Eastern Cooperative Oncology Group performance status.

Supplementary Table 3. A summary of Un-imputed patients’ baseline characteristics by study

| **Variable** | **Total No. 1,804** | **CASTOR No. 498** | **MAIA No. 737** | **POLLUX No. 569** | **P-value** |
| --- | --- | --- | --- | --- | --- |
| **Arm of the clinical study** | | | | | < 0.001 |
| Bortezomib and dexamethasone | 247 (14%) | 247 (50%) | 0 (0%) | 0 (0%) |  |
| Daratumumab plus bortezomib and dexamethasone | 251 (14%) | 251 (50%) | 0 (0%) | 0 (0%) |  |
| Daratumumab plus lenalidomide and dexamethasone | 654 (36%) | 0 (0%) | 368 (50%) | 286 (50%) |  |
| Lenalidomide and dexamethasone | 652 (36%) | 0 (0%) | 369 (50%) | 283 (50%) |  |
| **Age (years)** | 66 (58 - 72) | 58 (48 - 68) | 72 (64 - 72) | 65 (59 - 71) | < 0.001 |
| **Sex** | | | | | 0.029 |
| Male | 1,005 (56%) | 284 (57%) | 384 (52%) | 337 (59%) |  |
| Female | 799 (44%) | 214 (43%) | 353 (48%) | 232 (41%) |  |
| **Weight (kg)** | | | | | 0.002 |
| Median (IQR) | 74 (64 - 85) | 76 (67 - 88) | 72 (63 - 84) | 73 (62 - 83) |  |
| Missing | 326 (18%) | 39 (8%) | 0 (0%) | 287 (50%) |  |
| **Race** | | | | | < 0.001 |
| White | 1,503 (83%) | 435 (87%) | 675 (92%) | 393 (69%) |  |
| Asian | 128 (7%) | 23 (5%) | 5 (1%) | 100 (18%) |  |
| Black or African American | 64 (4%) | 20 (4%) | 28 (4%) | 16 (3%) |  |
| Other | 25 (1%) | 9 (2%) | 13 (2%) | 3 (1%) |  |
| Missing | 84 (5%) | 11 (2%) | 16 (2%) | 57 (10%) |  |
| **Proton Pump Inhibitor (Y/N)** | 577 (32%) | 143 (29%) | 264 (36%) | 170 (30%) | 0.014 |
| **ISS disease stage** | | | | | < 0.001 |
| I | 672 (37%) | 194 (39%) | 201 (27%) | 277 (49%) |  |
| II | 692 (38%) | 194 (39%) | 319 (43%) | 179 (31%) |  |
| III | 440 (24%) | 110 (22%) | 217 (29%) | 113 (20%) |  |
| **ECOGPS** | | | | | < 0.001 |
| 0 | 761 (42%) | 222 (45%) | 250 (34%) | 289 (51%) |  |
| 1 | 862 (48%) | 243 (49%) | 365 (50%) | 254 (45%) |  |
| ≥2 | 180 (10%) | 32 (6%) | 122 (17%) | 26 (5%) |  |
| Missing | 1 (<1%) | 1 (<1%) | 0 (0%) | 0 (0%) |  |
| **Comorbidity count** | | | | | < 0.001 |
| Median (IQR) | 5 (3 - 7) | 4 (2 - 6) | 6 (4 - 8) | 4 (3 - 6) |  |
| Missing | 24 (1%) | 12 (2%) | 1 (<1%) | 11 (2%) |  |
| **Gastrointestinal disorders** | 765 (42%) | 157 (32%) | 380 (52%) | 228 (40%) | < 0.001 |
| **Adverse events (grade ≥3)** | 1,469 (81%) | 347 (70%) | 681 (92%) | 441 (78%) | < 0.001 |

Data are median (IQR) or number of patients (%). P values per Chi-Square test for categorical data and Kruskal-Wallis test for continuous data. ISS Stage: international staging system (ISS) stage, ECOGPS: Eastern Cooperative Oncology Group performance status.

Supplementary Table 4. The top 10 grade ≥3 adverse events by study

| **Variable** | **Total No. 1,804** | **CASTOR No. 498** | **MAIA No. 737** | **POLLUX No. 569** | **P-value** |
| --- | --- | --- | --- | --- | --- |
| Neutropenia grade ≥3 | 634 (35%) | 43 (9%) | 338 (46%) | 253 (44%) | < 0.001 |
| Thrombocytopenia grade ≥3 | 336 (19%) | 190 (38%) | 69 (9%) | 77 (14%) | < 0.001 |
| Anaemia grade ≥3 | 331 (18%) | 80 (16%) | 156 (21%) | 95 (17%) | 0.035 |
| Pneumonia grade ≥3 | 202 (11%) | 44 (9%) | 112 (15%) | 46 (8%) | < 0.001 |
| Lymphopenia grade ≥3 | 156 (9%) | 29 (6%) | 102 (14%) | 25 (4%) | < 0.001 |
| Hypokalaemia grade ≥3 | 107 (6%) | 9 (2%) | 84 (11%) | 14 (2%) | < 0.001 |
| Leukopenia grade ≥3 | 92 (5%) | 10 (2%) | 66 (9%) | 16 (3%) | < 0.001 |
| Fatigue grade ≥3 | 94 (5%) | 19 (4%) | 49 (7%) | 26 (5%) | 0.063 |
| Diarrhoea grade ≥3 | 90 (5%) | 12 (2%) | 54 (7%) | 24 (4%) | < 0.001 |
| Cataract grade ≥3 | 89 (5%) | 1 (<1%) | 79 (11%) | 9 (2%) | < 0.001 |

Data are median (IQR) or number of patients (%). P values per Chi-Square test for categorical data and Kruskal-Wallis test for continuous data.

Supplementary table 5. A summary of patients’ baseline characteristics by treatment arms

| **Variable** | **Total No. 1,804** | **DRd No. 654** | **DVd No. 251** | **Rd No. 652** | **Vd No. 247** | **P-value** |
| --- | --- | --- | --- | --- | --- | --- |
| **Arm of the clinical study** | | | | | | < 0.001 |
| Bortezomib and dexamethasone | 247 (14%) | 0 (0%) | 0 (0%) | 0 (0%) | 247 (100%) |  |
| Daratumumab plus bortezomib and dexamethasone | 251 (14%) | 0 (0%) | 251 (100%) | 0 (0%) | 0 (0%) |  |
| Daratumumab plus lenalidomide and dexamethasone | 654 (36%) | 654 (100%) | 0 (0%) | 0 (0%) | 0 (0%) |  |
| Lenalidomide and dexamethasone | 652 (36%) | 0 (0%) | 0 (0%) | 652 (100%) | 0 (0%) |  |
| **Age (years)** | 66 (58 - 72) | 69 (64 - 72) | 58 (48 - 68) | 69 (64 - 72) | 58 (58 - 68) | < 0.001 |
| **Sex** | | | | | | 0.63 |
| Male | 1,005 (56%) | 362 (55%) | 137 (55%) | 359 (55%) | 147 (60%) |  |
| Female | 799 (44%) | 292 (45%) | 114 (45%) | 293 (45%) | 100 (40%) |  |
| **Weight (kg)** | 73 (63 - 85) | 73 (63 - 85) | 77 (67 - 88) | 71 (61 - 84) | 76 (66 - 87) | < 0.001 |
| Race | | | | | | 0.040 |
| White | 1,516 (84%) | 551 (84%) | 216 (86%) | 529 (81%) | 220 (89%) |  |
| Asian | 132 (7%) | 57 (9%) | 12 (5%) | 51 (8%) | 12 (5%) |  |
| Black or African American | 83 (5%) | 25 (4%) | 14 (6%) | 35 (5%) | 9 (4%) |  |
| Other | 73 (4%) | 21 (3%) | 9 (4%) | 37 (6%) | 6 (2%) |  |
| **ECOGPS** | | | | | | 0.045 |
| 0 | 761 (42%) | 266 (41%) | 106 (42%) | 273 (42%) | 116 (47%) |  |
| 1 | 863 (48%) | 314 (48%) | 132 (53%) | 305 (47%) | 112 (45%) |  |
| ≥2 | 180 (10%) | 74 (11%) | 13 (5%) | 74 (11%) | 19 (8%) |  |
| **ISS disease stage** | | | | | | 0.76 |
| I | 672 (37%) | 235 (36%) | 98 (39%) | 243 (37%) | 96 (39%) |  |
| II | 692 (38%) | 256 (39%) | 94 (37%) | 242 (37%) | 100 (40%) |  |
| III | 440 (24%) | 163 (25%) | 59 (24%) | 167 (26%) | 51 (21%) |  |
| **Proton Pump Inhibitor (Y/N)** | 577 (32%) | 219 (33%) | 72 (29%) | 215 (33%) | 71 (29%) | 0.33 |
| **Comorbidity count** | 5.0 (3.0 - 7.0) | 5.0 (4.0 - 7.0) | 4.0 (2.0 - 5.0) | 5.0 (4.0 - 7.0) | 4.0 (2.9 - 6.0) | < 0.001 |
| **Gastrointestinal disorders** | 765 (42%) | 313 (48%) | 79 (31%) | 295 (45%) | 78 (32%) | < 0.001 |
| **Adverse events (grade ≥3)** | 1,469 (81%) | 588 (90%) | 192 (76%) | 534 (82%) | 155 (63%) | < 0.001 |

Data are median (IQR) or number of patients (%). P values per Chi-Square test for categorical data and Kruskal-Wallis test for continuous data. ISS Stage: international staging system (ISS) stage, ECOGPS: Eastern Cooperative Oncology Group performance status. DRd: Daratumumab, Lenalidomide, and Dexamethasone. DVd: Daratumumab, Bortezomib, and Dexamethasone. Rd: Lenalidomide and Dexamethasone. Vd: Bortezomib and Dexamethasone


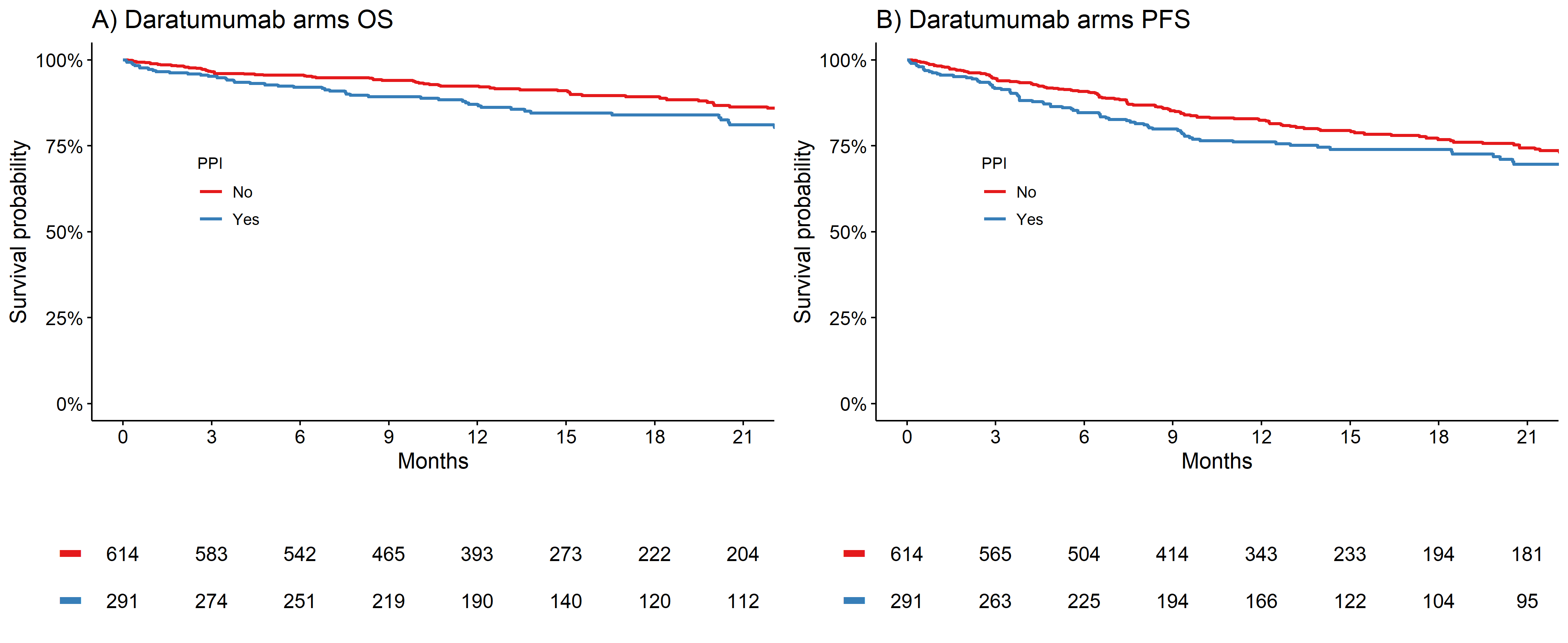


Supplementary Figure 1: Kaplan-Meier curves for proton pump inhibitor use and survival outcomes by daratumumab arms.


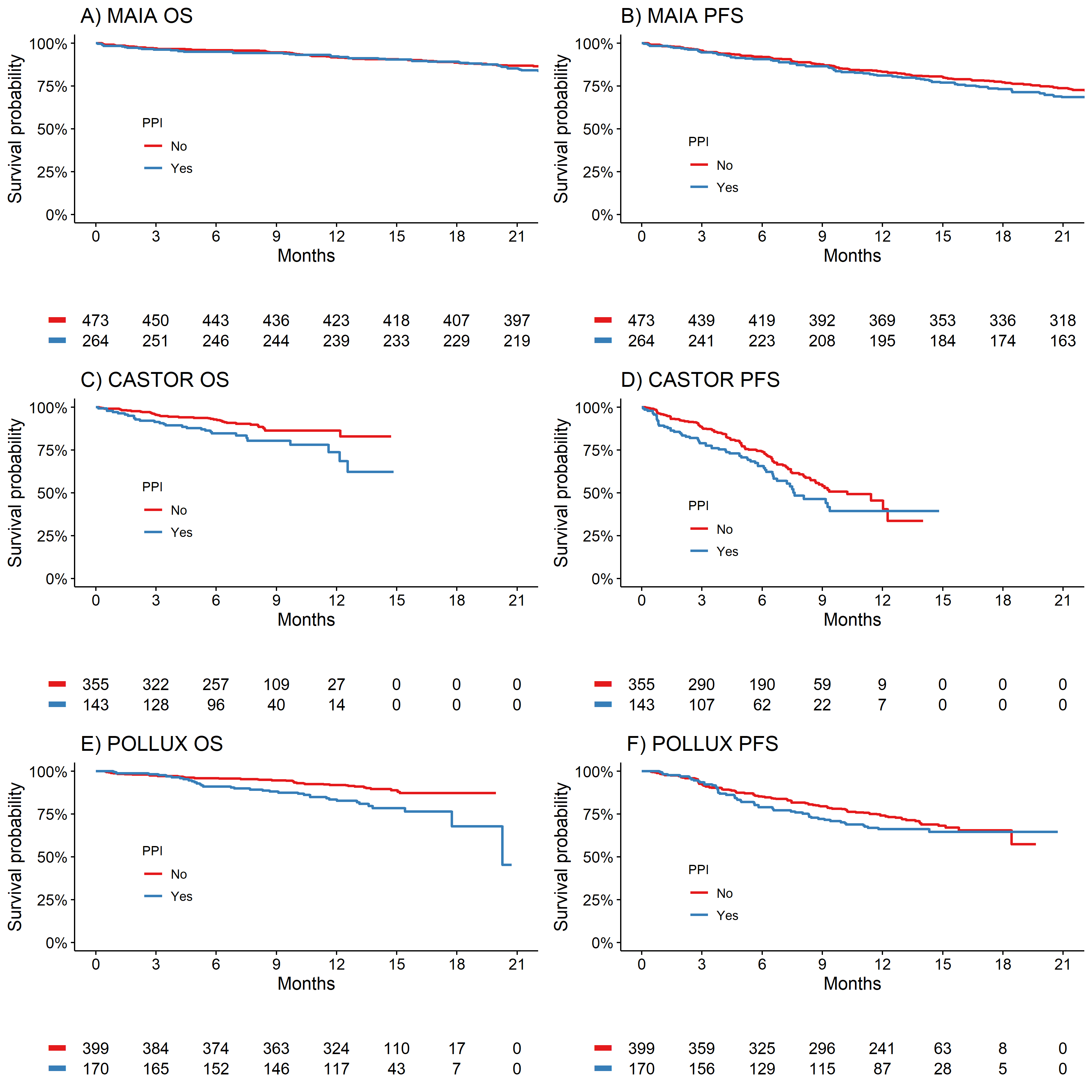


Supplementary Figure 2: Kaplan-Meier curves for proton pump inhibitor use and survival outcomes by study.

# References

1. Ludwig H, Durie BG, Bolejack V, Turesson I, Kyle RA, Blade J, Fonseca R, Dimopoulos M, Shimizu K, San Miguel J, Westin J. Myeloma in patients younger than age 50 years presents with more favorable features and shows better survival: an analysis of 10 549 patients from the International Myeloma Working Group. Blood, The Journal of the American Society of Hematology. 2008 Apr 15;111(8):4039-47.
2. Qian J, Jin J, Luo H, Jin C, Wang L, Qian W, Meng H. Analysis of clinical characteristics and prognostic factors of multiple myeloma: a retrospective single-center study of 787 cases. Hematology. 2017 Sep 14;22(8):472-6.
3. Boyd KD, Ross FM, Chiecchio L, Dagrada G, Konn ZJ, Tapper WJ, Walker BA, Wardell CP, Gregory WM, Szubert AJ, Davies FE. Gender disparities in the tumor genetics and clinical outcome of multiple myeloma. Cancer epidemiology, biomarkers & prevention. 2011 Aug 1;20(8):1703-7.
4. Ailawadhi S, Jacobus S, Sexton R, Stewart AK, Dispenzieri A, Hussein MA, Zonder JA, Crowley J, Hoering A, Barlogie B, Orlowski RZ. Disease and outcome disparities in multiple myeloma: exploring the role of race/ethnicity in the Cooperative Group clinical trials. Blood cancer journal. 2018 Jul 6;8(7):67.
5. Derman BA, Langerman SS, Maric M, Jakubowiak A, Zhang W, Chiu BC. Sex differences in outcomes in multiple myeloma. British journal of haematology. 2021 Feb;192(3):e66.
6. Shah UA, Whiting K, Devlin S, Ershler R, Kanapuru B, Lee DJ, Tahri S, Gwise T, Rustad EH, Mailankody S, Lesokhin AM. Extreme body mass index and survival in newly diagnosed multiple myeloma patients. Blood Cancer Journal. 2023 Jan 12;13(1):13.
7. Wang B, Derman BA, Langerman S, Stepniak E, Johnson J, Zhang W, Jakubowiak AJ, Chiu BC. Body mass index and overall survival in multiple myeloma.
8. Beason TS, Chang SH, Sanfilippo KM, Luo S, Colditz GA, Vij R, Tomasson MH, Dipersio JF, Stockerl‐Goldstein K, Ganti A, Wildes T. Influence of body mass index on survival in veterans with multiple myeloma. The oncologist. 2013 Oct 1;18(10):1074-9.
9. Schmidt TM. High or low? Assessing disease risk in multiple myeloma. Hematology. 2022 Dec 9;2022(1):349-55.
10. Afram G, Gran C, Borg Bruchfeld J, Wagner AK, Hussain A, Alici E, Nahi H. Impact of performance status on overall survival in patients with relapsed and/or refractory multiple myeloma: Real‐life outcomes of daratumumab treatment. European Journal of Haematology. 2020 Aug;105(2):196-202.
11. Wildes T, Luo S, Colditz GA, Carson KR. Comorbidities impact survival in multiple myeloma: Analysis of the Veterans Health Administration national database. Blood. 2012 Nov 16;120(21):760.
12. Sverrisdóttir IS, Rögnvaldsson S, Thorsteinsdottir S, Gíslason GK, Aspelund T, Turesson I, Björkholm M, Gregersen H, Hveding Blimark C, Landgren O, Kristinsson SY. Comorbidities in multiple myeloma and implications on survival: a population‐based study. European journal of haematology. 2021 Jun;106(6):774-82.
13. Gregersen H, Vangsted AJ, Abildgaard N, Andersen NF, Pedersen RS, Frølund UC, Helleberg C, Broch B, Pedersen PT, Gimsing P, Klausen TW. The impact of comorbidity on mortality in multiple myeloma: a Danish nationwide population‐based study. Cancer medicine. 2017 Jul;6(7):1807-16.
